# Interprofessional education at medical faculties in German-speaking countries – institutional challenges and enablers of successful curricular implementation: A mixed-methods study

**DOI:** 10.3205/zma001769

**Published:** 2025-09-15

**Authors:** Carlos González Blum, Robert Richter, Ursula Walkenhorst

**Affiliations:** 1 Osnabrück University, FB 8 – Human Sciences, Institute of Health Research and Education (IGB), Osnabrück, Germany; 2 Furtwangen University, Faculty of Health, Medical and Life Sciences, Furtwangen, Germany

**Keywords:** interprofessional education, curricular implementation, medical curriculum, stakeholders

## Abstract

**Study aim:**

Interprofessional education (IPE) is necessary in the training of health care professionals in preparation for future teamwork. However, sustainable integration into the medical curriculum is currently restricted by heterogeneous legal regulations. This study examines the factors and conditions upon which the implementation of IPE in medical education in German-speaking countries is contingent.

**Methods:**

A mixed-methods, cross-sectional approach was carried out. This included a quantitative data collection of established IPE courses (type of course of study and IPE), which was evaluated descriptively. A qualitative approach encompassed semi-structured interviews with experts at medical faculties in German-speaking countries. In addition, stakeholder analyses were conducted.

**Results:**

Study results demonstrated that enablers of curricular implementation of IPE include structural and logistical factors, as well as legal frameworks. Other important factors include high interest and involvement of various stakeholders, investment in resources, and utilization of various strategies to address curricular requirements. Challenges for sustainable implementation include dealing with a strict curricular structure, low stakeholder motivation, and low prioritization of IPE. Recommendations for addressing these challenges were specified. The stakeholder analyses revealed distinct constellations of stakeholders across the institutions examined.

**Conclusion:**

Legal regulations and licensing requirements, logistical and structural factors, as well as a creative approach to address curricular requirements play a crucial role in currently implemented IPE in German-speaking countries. Stakeholders with a strong interest in and understanding of IPE are fundamental to its success.

## Zusammenfassung

**Zielsetzung:**

Interprofessionelle Lehre (IPL) ist in den Ausbildungen der Gesundheitsberufe notwendig, um das Personal auf künftige gemeinsame Arbeit vorzubereiten. Eine nachhaltige Integration in das humanmedizinische Curriculum wird jedoch derzeit unter anderem durch heterogene gesetzliche Regularien erschwert. Die vorliegende Studie untersucht die Faktoren und Rahmenbedingungen, von denen die Verstetigung von IPL in der Hochschulmedizin im deutschsprachigen Raum abhängig ist.

**Methodik:**

Es wurde ein Mixed-Methods-Querschnitt-Ansatz gewählt. Dieser umfasste eine quantitative Datenerhebung von verstetigten IPL-Angeboten (Art des Studiengangs und der IPL) die deskriptiv ausgewertet wurde, sowie einen qualitativen Zugang mittels teilstrukturierter Leitfadeninterviews mit Expert:innen an humanmedizinischen Fakultäten im deutschsprachigen Raum. Darauf aufbauend wurde eine Stakeholder-Analyse durchgeführt.

**Ergebnisse:**

Die Studie ergab, dass Gelingensfaktoren strukturelle und logistische Faktoren sowie gesetzliche Rahmenbedingungen beinhalten. Weitere Faktoren sind ein hohes Interesse und Einbindung verschiedener Stakeholder, Investitionen in Ressourcen und verschiedene Strategien im Umgang mit curricularen Voraussetzungen. Zu den Herausforderungen der Verstetigung gehören der Umgang mit der strikt vorgegebenen curricularen Struktur, geringe Stakeholder-Motivation und Priorisierung von IPL. Empfehlungen zum Umgang mit diesen wurden spezifiziert. Die Stakeholder-Analysen zeigen, dass die Konstellationen von Stakeholdern an den analysierten Institutionen unterschiedlich sind.

**Schlussfolgerung:**

Gesetzliche, logistische und strukturelle Faktoren sowie ein kreativer Umgang mit curricularen Vorgaben spielen eine entscheidende Rolle in der aktuell verstetigten interprofessionellen Lehre im deutschsprachigen Raum. Stakeholder mit starkem Interesse und Verständnis für IPL sind fundamental für den Erfolg.

## 1. Background

Interprofessional (IP) teamwork among healthcare professionals is widely regarded as a key strategy for addressing future health care demands [1], [2], [3]. It has been found to lead to an improvement of health care outcomes [4], [5], more efficient use of resources [6], [7], and increased job satisfaction [8]. Interprofessional education (IPE) is considered one of the most promising solutions to prepare healthcare professionals for collaborative work [2], [9]. Interprofessional education takes place when two or more professional groups learn about, from and with each other for the purpose of improving teamwork and the quality of future healthcare [2], [https://www.caipe.org/]. Competency frameworks, for example the Interprofessional Education Collaborative (IPEC) [10], have developed core competencies for IPE.

### 1.1. Current state of research

Awareness of the importance of IPE has increased in German-speaking countries [11], [12]. In Switzerland, favorable health policies for IP training in the health professions have played an important role [11]. Furthermore, in 2017, the Swiss Federal Office of Public Health began funding programs for IP research projects [13]. Among current IP courses, IP “education days” designed by students from various health professions are carried out [14]. Since the 2005 health care reform, Austrian legislators have also increasingly supported IP approaches in both healthcare delivery and professional training [12]. As a result, current Austrian training regulations allow the integration of IPE. For example, a new IPE course between the Medical Faculty of the Johannes Kepler University Linz and the University of Applied Sciences for Health Professions Upper Austria, in which students from nine different healthcare professions participate in a joint course on IP collaboration and IP job shadowing, has shown promising results [15]. In Germany, a key factor was the funding of numerous IPE projects by the Robert Bosch Foundation (RBS) [16], which enabled the development and implementation of these initiatives [17], [18], [19], [20]. However, many IPE courses for medical students are often voluntary, despite being integrated into the curriculum. For students of other professions, such as nursing or physiotherapy, with whom medical students interact, course attendance is often mandatory [21], [22]. One of the most significant challenges in integrating an IPE course into the curriculum is the absence of legal regulations that effectively and sustainably embed IP teaching and learning in the training of all health professions [12], [23]. 

Beyond legal conditions and project funding, it remains unclear which additional factors are essential to establishing IPE. A study [24] examined 152 surveys from several academic institutions in 45 countries, including Germany and Switzerland. The survey explored both enabling factors and challenges related to IPE. Some of the enablers included dedicated staff and support from institutional management, e.g. faculty leadership and the Dean of Studies. One challenge was a lack of understanding of IPE among faculty members. Due to the low survey participation of German and Swiss institutions, it is difficult to draw conclusions about the specific situation in these countries. However, the analysis offers the perspective that the enablers and challenges related to IPE are not limited to legal regulations and financial factors.

### 1.2. Theoretical background

Experts at German medical faculties consider a mandatory implementation of IPE through compressing or removing of other teaching content impossible due to the requirements of the current Medical Licensing Regulations (ÄApprO) [25]. This represents a significant obstacle to the integration of IPE into medical curricula, which can be explained by Institutional Theory (IT). Institutional Theory describes that organizations comply with rules and requirements to gain legitimacy and support [26]. While IT can provide insight into what influences a faculty at the institutional level to maintain regulatory standards, the theory does not address individuals or stakeholders, their self-interest and power, or the internal dynamics of an institution [27]. Stakeholder Theory (ST), on the other hand, recognizes the existence of various stakeholders who can either influence or be influenced by an organization [28]. Furthermore, Freeth & Reeves, in their 3P model, emphasize that establishing an IPE course requires not only legal regulations but also faculty development that supports all involved stakeholders [29]. This is considered crucial for the sustainability of an IPE program [30], [31], [32].

Currently, there is no comprehensive overview of where IPE is integrated in the medical curriculum in German-speaking countries. Enabling factors contributing to the integration of IPE in German-speaking medical schools – as well as perceived barriers, challenges that have been overcome, and current challenges – have not yet been systematically studied.

#### 1.2.1. Research question

Upon which factors and conditions is the curricular implementation of IPE in medical education in German-speaking countries contingent?

## 2. Methods

The qualitative part of this study follows the Standards for Reporting Qualitative Research [33].

To address the research question, a mixed-methods, cross-sectional design was employed, consisting of three consecutive analyses. First, a quantitative data collection (*analysis 1*) of implemented IPE courses was carried out. Subsequently, qualitative interviews (*analysis 2*) and stakeholder analyses (*analysis 3*) were conducted. 

**Figure 1:**
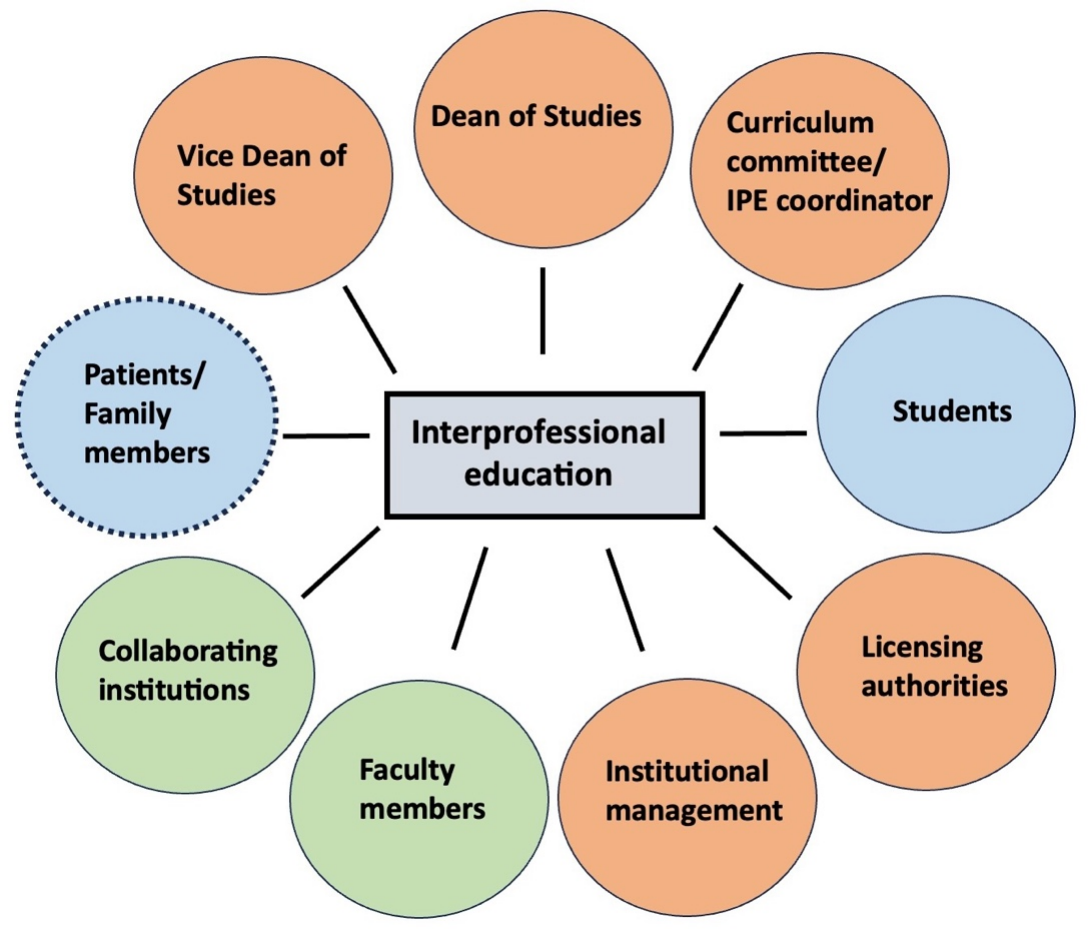
Stakeholders for interprofessional education The figure illustrates stakeholders who either influence or are influenced by IPE. Orange indicates stakeholders who primarily influence IPE; blue indicates stakeholders who are primarily influenced by IPE; green indicates stakeholders who both influence and are influenced by IPE; The dotted line underscores that patients and their families are the core focus of efforts to provide optimal healthcare.

To identify implemented IPE courses, a document search (*analysis 1*) was conducted for all medical faculties in Germany, Switzerland, and Austria (DACH countries). The collected information (type of course of study and IPE) was available online from study and examination regulations and module descriptions. To confirm that the relevant documents found in the general search were up to date and complete, subsequent contact was made via email or telephone with the dean's office or IPE representative. The data were analyzed descriptively (absolute frequency and percentage). For *analysis 2*, semi-structured expert interviews were conducted according to Meuser and Nagel [34], and these were evaluated using qualitative content analysis according to Kuckartz [35]. The analyses are divided into two steps (2a and 2b). *Analysis 2a* is a category-based analysis for the key category enabling factors. *Analysis 2b* shows a multidimensional category analysis. A concept map (see attachment 1 ) illustrates the relationships between the categories. The stakeholder analyses (*analysis 3*) represent the constellations of stakeholders at the institutions (see figure 1 (Fig. 1)) involved in the IPE coursework. Stakeholders were classified according to their position of power based on decision-making authority in the curriculum (see attachment 2 ) and according to their interest in establishing IPE in the curriculum. Stakeholders with high positions of power were defined as individuals who have decision-making authority based on resource allocation and those with the ability to directly influence project implementation [36]. This includes, for example, a dean’s office or vice dean's office [37], but also a curriculum committee or the institutional management of a collaborating institution [38], for example from another participating profession.

### 2.1. Sampling

The explicit sampling method is deductive. Experts from medical institutions with established IPE were recruited if they served as project coordinators or contact persons responsible for the course. Theoretical saturation [39] for the number of expert interviews was considered reached when most of the stakeholders defined in figure 1 (Fig. 1) were mentioned by interviewees. 

### 2.2. Recruitment method/field access

Institutions considered for recruitment offered established IPE courses. These could be either part of the core curriculum or as a sustainable elective course in a bachelor’s or master’s degree program, as a regular or model medical degree program. A regular medical program provides medical training within a standard degree curriculum; a model medical program is governed by §41 of the ÄApprO. Once the inclusion criteria were met, experts were contacted via email. A short questionnaire to collect personal data was completed, and a consent form to conduct the interview was signed by the interviewee. A pre-test interview was conducted with an expert in IPE.

### 2.3. Data collection and analysis

The theme-centered interview guide (see attachment 3 ) included theory-guided, deductive main questions and topic-specific follow-up questions [34]. Reflexivity was considered in various ways: Before conducting the interviews, a preliminary understanding of each interview question was discussed amongst authors within the author team. In addition, a research diary was kept to make the research process retrospectively transparent and accessible for critical evaluation. A postscript was written following each online interview. The interviews were transcribed verbatim using f4transkript software. Qualitative content analysis according to Kuckartz (see attachment 4 ) [35] was chosen as the analysis method. This approach is grounded in constructivist, iterative, and reflexive epistemological assumptions. The interviews were analyzed using f4analyse software.

To ensure high coding quality, a discursive data interpretation was conducted by two researchers. Intercoder agreement (kappa) was calculated using MAXQDA software.

## 3. Results

### 3.1. Analysis 1: Descriptive analysis of established IPE courses in DACH countries

The document search was carried out between March 3 and July 1, 2023. Of the 44 medical degree programs in Germany, 30 follow the regular curriculum and 14 are model courses of study (see table 1 (Tab. 1)). IPE is implemented into the curriculum in 53% of regular medical programs and 91% of model programs. In regular curricular programs, twice as many elective IP courses are offered compared to IP courses integrated in the core curriculum. In the model medical programs, the number of IP courses in the core curriculum and IP elective courses is the same (see table 2 (Tab. 2)). All German-speaking medical faculties in Switzerland offer IPE that is implemented in the curriculum. Of four medical faculties in Austria, one degree program offers an IPE course that is implemented into the curriculum (see table 3 (Tab. 3)).

#### 3.1.1. Intercoder agreement

Intercoder agreement was 71% (Kappa=0.70) before and 97.7% (Kappa=0.98) after discussion and revision.

### 3.2. Analysis 2a: Category-based analysis of enabling factors across all institutions 

A total of eight interviews were conducted. Apart from the licensing authorities, all defined stakeholders were mentioned. Based on the researchers’ assessment, theoretical saturation was thus achieved.

The enabling factors shared by interviewees were categorized as similarities, differences, and notable findings.

**Figure 2:**
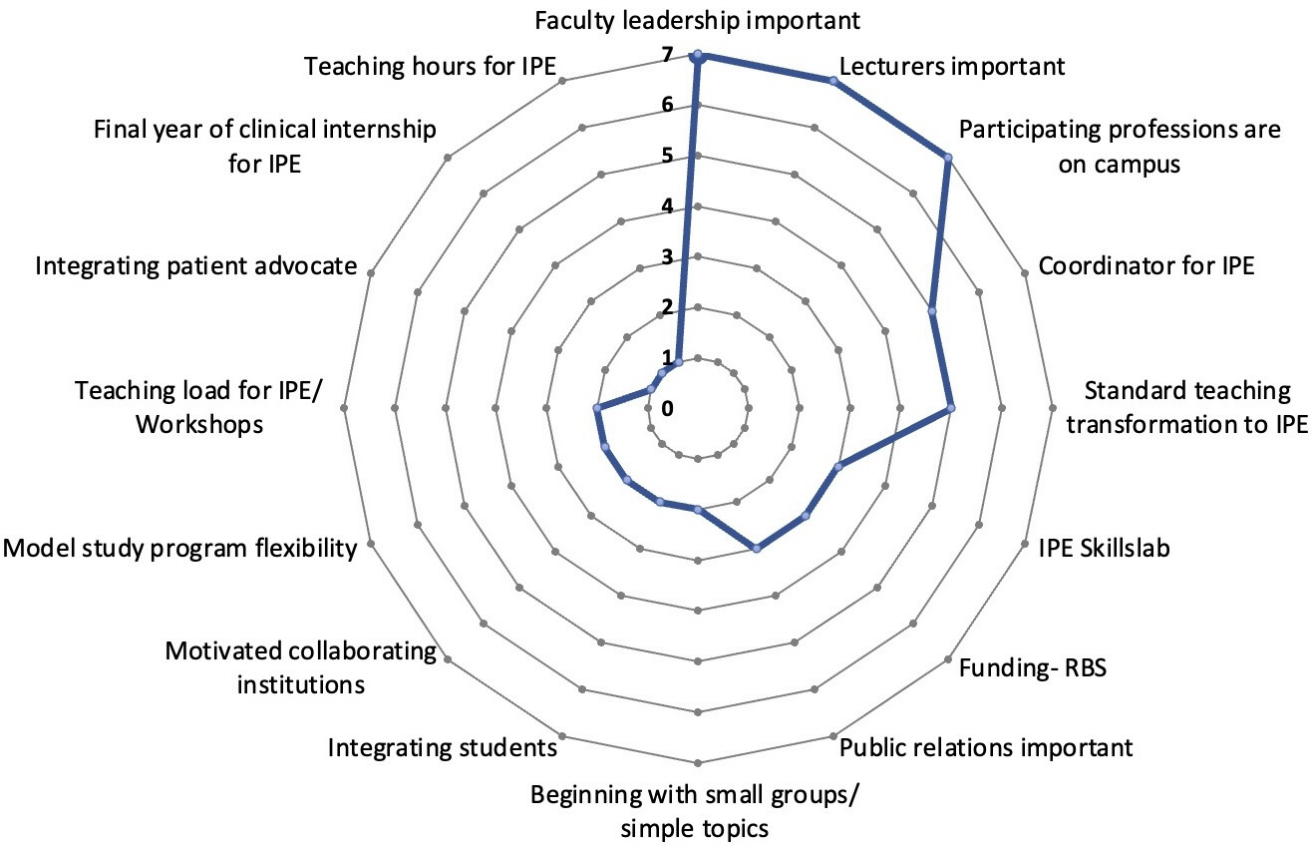
Frequencies of enabling factors The frequencies of the enabling factors reported by the experts surveyed are illustrated by a radar diagram. All enabling factors mentioned by each of the eight experts interviewed were included in the analysis.

- *Similarities:* 16 factors played a crucial role in enabling the IPE course. A radar diagram illustrates the frequencies of enabling factors (see figure 2 (Fig. 2)).
- *Differences: *The interviewed expert from the Swiss faculty summarized the legal and regulatory requirements regarding IPE in Switzerland with the following statement:* “The legal requirements are already in place. Everyone has to do it, and they can only choose how they do it.”* (D, paragraph 16). According to one model medical program (E), being a model program is not in itself an enabler. This view contrasts with the perspectives of interviewees at the other model study programs (A, G), who shared that they have a certain degree of curricular flexibility.
- *Notable findings: *The Swiss faculty and all three model medical programs reported that standard teaching or training is often transformed to IPE. This was mentioned by only one of four interviewees at regular courses of medical study.

### 3.3. Analysis 2b: Multidimensional category analysis

The multidimensional analysis (see attachment 5 ) consists of paraphrased interview statements. According to the analysis, the sustainable implementation of IPE is a prerequisite for ensuring the best possible future healthcare. For Switzerland, this has already been established. Many medical faculties in Germany, however, are waiting until IPE is required by medical licensing regulations before initiating efforts for curricular implementation. Institutions that currently offer IPE employ and recommend strategies such as transforming standard mono-professional teaching into IPE, and some use the final year of clinical internship to implement IPE courses. Support from faculty leadership is considered essential, while its absence is viewed as a barrier. Also motivated lecturers help to advance IPE. However, they may also be critical or skeptical of it, posing a potential obstacle. A lack of support for IPE initiatives necessitates educational and persuasive efforts, including practical examples that demonstrate IPE as essential preparation for future high-quality healthcare delivery. Project evaluations and publications could be helpful for this. The inclusion of new, motivated collaborators and stakeholders, including students, can be highly beneficial.

### 3.4. Analysis 3: Stakeholder analyses 

Eight stakeholder analyses were conducted (see attachment 6 ). No constellation of stakeholders at the respective institutions was identical to another. Furthermore, no single interviewed expert at an institution named all the defined stakeholders. In most institutions, there are stakeholders with both high and low power and a high interest in IPE (A, B, C, E, F, G). However, other analyses demonstrated multiple stakeholders with high power and varying interest (B), low power and varying interest (F), and one institution in which a stakeholder with high power has low interest in IPE (H).

## 4. Discussion

Among the most influential factors affecting IPE are legal regulations and licensing requirements [29]. In Switzerland, accreditation requirements are the most important enablers. The conceptual framework PROFILES (Principal Relevant Objectives and Framework for Integrative Learning and Education in Switzerland) [40] is strongly based on the CanMEDS model [https://www.profilesmed.ch/], which emphasizes the integral role of Collaborator. In the absence of legal licensing requirements for IPE, German faculties employ strategies to implement it. More than half of all interviewees reported that standard mono-professional teaching at their institutions is transformed into IPE. The expert commission for the Master Plan 2020 also made this recommendation: *“In medical training, there are a multitude of elements that can be connected to interprofessionalism. These can be focused interprofessionally without significant additional effort.”* [40]

According to most expert interviews, strong stakeholder support and understanding of IPE plays a crucial role. In Khalili’s study [24], committed staff was also described as an important enabling factor. Moreover, since faculty members are considered enablers of effective IPE, faculty development has also been described as important [13], [30]. Faculty development provides tools for developing behaviors for IP teaching contexts [41], improves IP learning, and has a positive impact on learning outcomes [42]. Freeth and Reeves’ 3P model also highlights lecturers’ experience and enthusiasm as relevant factors for IPE formats [29]. However, the model does not address the role of faculty leadership. In contrast, Khalili emphasizes leadership support as a key factor in the success of IPE formats [24]. This is consistent with the findings of this study. Support from faculty leadership is essential, not least because it can help secure a project coordinator for IPE. The expansion of a successful IPE program requires top-down support and the investment of resources in staff, time, and money [37]. Furthermore, individuals in positions of authority typically have decision-making power that is based on resource allocation and setting institutional priorities [43]. Within the context of faculty development, faculty leadership can clearly and transparently convey the importance of IPE. This may be valuable in helping to challenge existing negative attitudes toward it [44], [45]. Since critics and skeptics of IPE were mentioned in several interviews, this may also be a relevant strategy to address this. It has been described that faculty development should take place prior to the initiation of IPE efforts [46], and should continue throughout the implementation of IPE programs [47]. Progress cannot be made if faculty leadership does not make IPE a priority [38]. Several interviewees explicitly mentioned that faculty leadership decided IPE should be a focus of their medical course of study. Despite an expert at one institution (H) describing that their IPE courses were currently stable, the lack of support and interest in IPE from faculty leadership could jeopardize the sustainability of existing IPE formats. Given the described importance of support of faculty leadership, as well as their significant decision-making authority, it might be useful to include them in the 3P model under *presage*. 

In one interview, it was noted that although an institution may consider IPE important, it is just one of many competing priorities (G, paragraph 56), such as digitalization or planetary health, which will also be included in the updated ÄApprO [48], [https://nklm.de/zend/menu]. In this context, competencies can complement one another. For example, learning objectives related to digital skills can be effectively addressed through an IP teaching format, e.g. to practice communication skills through patient apps or through the application of artificial intelligence systems [49]. Although the frequency of mentioned enabling factors should be considered, the factors themselves are neither more nor less important than the others. Thus, an enabler mentioned only once or twice has a similar “weight” to a more frequently mentioned factor, as these factors were crucial at the respective institutions. For example, two experts from different institutions noted that student demand for IPE was a significant driver that increased the pressure to act. The expert at institution H was the only one to mention the integration of a patient advocate. The institution lacks a project coordinator for IPE, and unlike other institutions, faculty leadership shows no discernible interest in IPE, setting it apart from the rest. According to the interviewed expert, the integration of a patient advocate, along with motivated faculty, a skills lab, and the co-location of participating health professions at the university forms a crucial constellation of factors that create a “collective power” enabling the IPE courses. A review demonstrated that faculty members are not the sole developers of IPE formats. Among 80 programs examined, 20% reported contributions from students, patients, and families [50]. 

Most German faculties surveyed are aware of the legal regulations and limitations. Several experts at these institutions also reported that other faculties without IPE formats most likely adhere to the curricular requirements of the current ÄApprO. These findings are consistent with the core principles of IT. The theory is similarly applicable to Swiss institutions, as these institutions also follow the legal requirement to offer IPE to fulfill accreditation requirements.

Stakeholder Theory describes that the success of an organization depends on the integration of multiple stakeholders [28]. All defined stakeholders were mentioned by the interviewed experts, except for the licensing authorities. This underscores and confirms the relevance of ST, as each stakeholder played an important role in the implementation of the IPE courses. 

It is important to recognize, however, that all stakeholders are interconnected. As described by Freeman: *if you think about it, it makes sense. All stakeholders are interdependent* [28].

### 4.1. Limitations

One of the limitations of this study is that not all medical faculties in the DACH countries were surveyed. No Austrian faculties were interviewed because the only eligible institution recently published its IPE results [15], making an anonymous interview impossible. Only medical faculties were surveyed. However, curricular inflexibility, particularly at medical faculties, has been described as a major barrier to the implementation of IPE [25]. Qualitative research carries inherent limitations due to its reliance on the researcher’s skills, interpretations, and potential biases [51], [52].

## 5. Conclusion

Legal regulations and licensing requirements, logistical and structural factors, as well as a creative approach to address curricular requirements play a crucial role in currently implemented IPE in German-speaking countries. Stakeholders with a strong interest in and understanding of IPE are fundamental to its success.

## Authors’ ORCIDs

- Carlos González Blum: [0000-0002-6853-3888]
- Robert Richter: [0000-0002-8644-765X]
- Ursula Walkenhorst: [0000-0003-4614-5478]

## Competing interests

The authors declare that they have no competing interests. 

## Supplementary Material

Concept map with main and subcategories

Stakeholders and their position of power based on decision-making authority

Interview guide

Qualitative content analysis according to Kuckartz

Multidimensional category analysis

Stakeholder analyses

## Figures and Tables

**Table 1:**
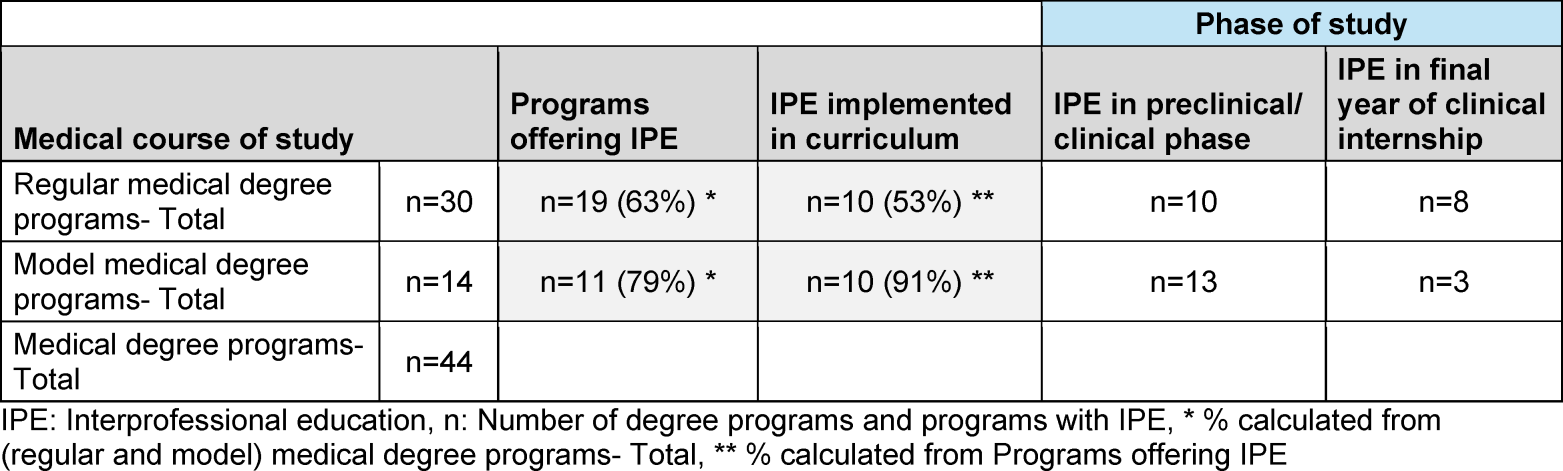
Medical degree programs with interprofessional education in Germany

**Table 2:**
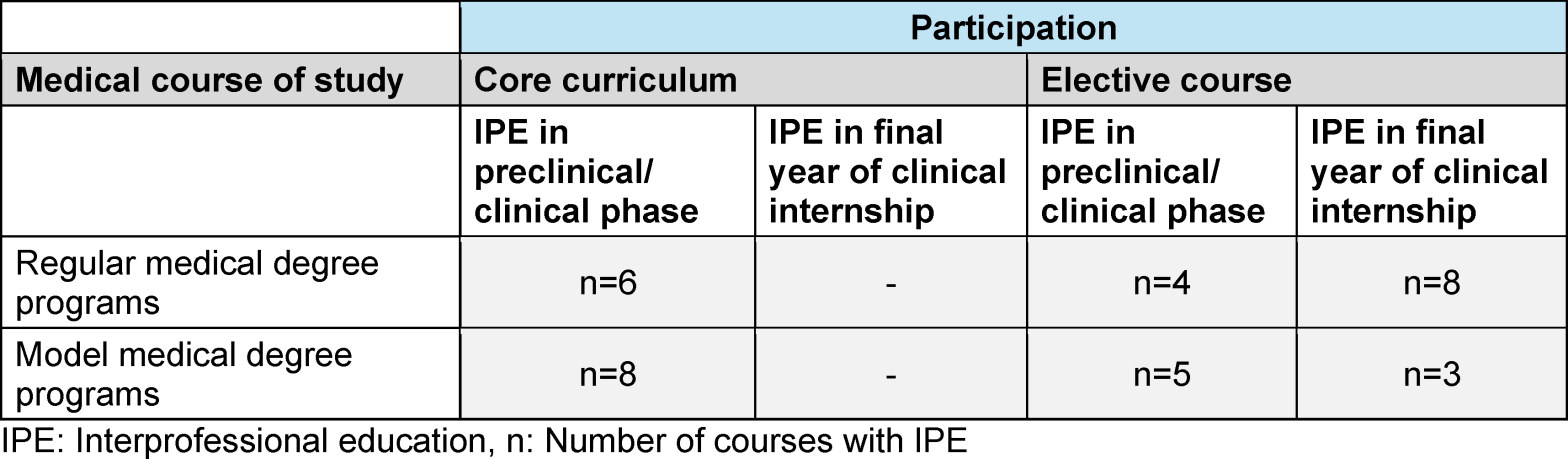
Interprofessional education courses in core and elective curricula of German medical degree programs

**Table 3:**
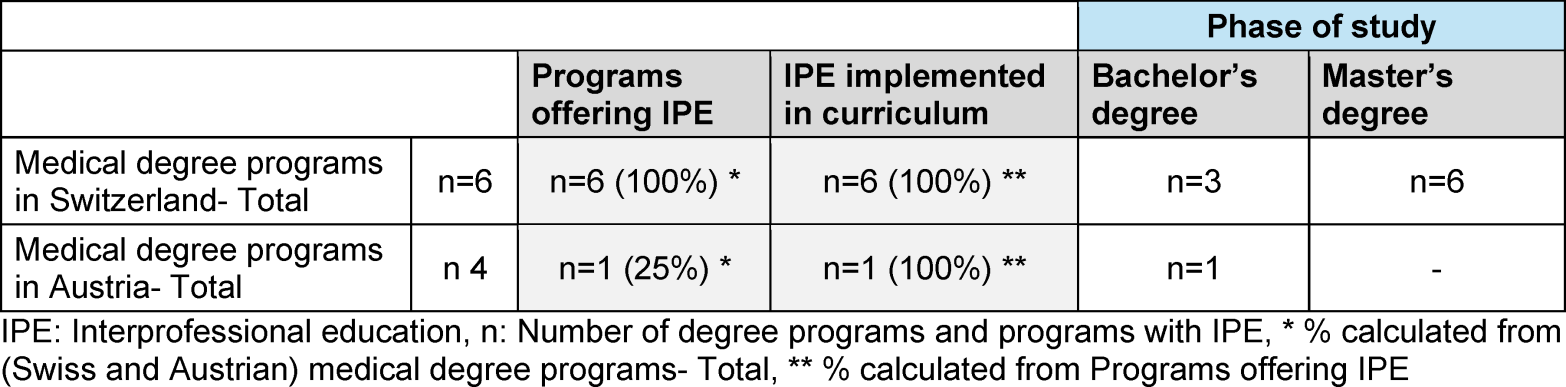
Medical degree programs with interprofessional education in Switzerland and Austria
