# Supplementary material for: Interprofessional education at medical faculties in German-speaking countries – institutional challenges and enablers of successful curricular implementation: A mixed-methods study
Source: GMS J Med Educ. 2025 Sep 15;42(4):Doc45. doi: 10.3205/zma001769 (PMC12527387; doi:10.3205/zma001769)
Supplement: Concept map with main and subcategories [file JME-42-45-s-001.pdf]

Attachment 1: Concept map with main and subcategories

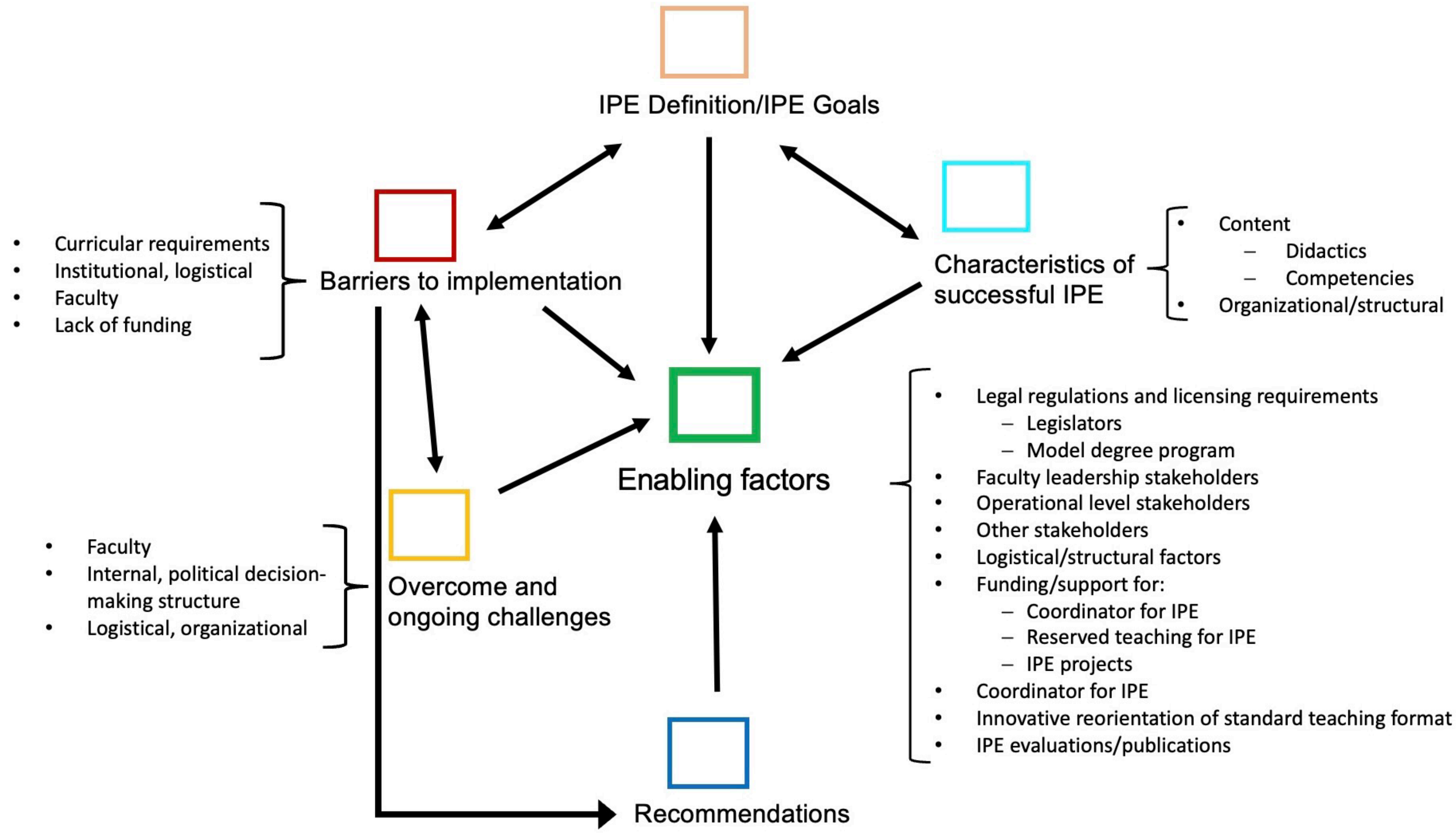

The concept map shows all main categories and subcategories. Arrows symbolize the direction of influence; the two-way arrow lines symbolize interrelationships.

Attachment 1 to González Blum C, Richter R, Walkenhorst U. *Interprofessional education at medical faculties in German-speaking countries – institutional challenges and enablers of successful curricular implementation: A mixed-methods study.* GMS J Med Educ. 2025;42(4):Doc45. DOI: 10.3205/zma001769
