# Supplementary material for: Interprofessional education at medical faculties in German-speaking countries – institutional challenges and enablers of successful curricular implementation: A mixed-methods study
Source: GMS J Med Educ. 2025 Sep 15;42(4):Doc45. doi: 10.3205/zma001769 (PMC12527387; doi:10.3205/zma001769)
Supplement: Stakeholders and their position of power based on decision-making authority [file JME-42-45-s-002.pdf]

Attachment 2: Stakeholders and their position of power based on decision-making authority

Stakeholders	Position of power/ decision-making authority
Dean/Vice Dean	High
Dean of Studies	High
Curriculum committee/ IPE coordinator	High
Institutional management	High
Lecturers	Low
Students	Low
Collaborating institutions	Low
Patients/family members	Low
Licensing authorities	Low

Stakeholders are classified according to their position of power based on decision-making authority in the curriculum

Attachment 2 to González Blum C, Richter R, Walkenhorst U. *Interprofessional education at medical faculties in German-speaking countries – institutional challenges and enablers of successful curricular implementation: A mixed-methods study.* GMS J Med Educ. 2025;42(4):Doc45. DOI: 10.3205/zma001769
