# Supplementary material for: Interprofessional education at medical faculties in German-speaking countries – institutional challenges and enablers of successful curricular implementation: A mixed-methods study
Source: GMS J Med Educ. 2025 Sep 15;42(4):Doc45. doi: 10.3205/zma001769 (PMC12527387; doi:10.3205/zma001769)
Supplement: Interview guide [file JME-42-45-s-003.pdf]

Attachment 3: Interview guide

Good afternoon, Ms./Mr. (Name),

Thank you very much for agreeing to the interview!
Just to give you an overview, the conversation is planned to last approximately 60 minutes. Following the interview, the data collected will be used anonymously. It will then no longer be possible to identify you personally.

Before we begin, do you agree to the recording of the conversation? We are not starting the recording yet. Do you have any questions before we begin?

Good, then I would like to start the recording now and start with the questions.

I would like to briefly explain the topic of the research project to you again: My research topic concerns the factors and conditions of interprofessional education in higher education. Your perspective is therefore very valuable to me.

First, a general question regarding interprofessional education:

Question 1: The call for interprofessional healthcare has gained momentum in recent years. The demand for interprofessional education in the healthcare sector has also become increasingly relevant. What does interprofessional education mean to you?

Follow-up questions:

- Can you think of anything else regarding this?
- Can you provide an example?

1b) What is the goal or what are the goals of interprofessional education?
1c) Which interprofessional formats and forms of interaction are you familiar with?

Attachment 3 to González Blum C, Richter R, Walkenhorst U. *Interprofessional education at medical faculties in German-speaking countries – institutional challenges and enablers of successful curricular implementation: A mixed-methods study.* GMS J Med Educ. 2025;42(4):Doc45. DOI: 10.3205/zma001769

Question 2: There is no universally accepted definition of successful interprofessional education. In your view, what defines a successful interprofessional education course?

Follow-up questions:

- Can you think of anything else regarding this?
- Can you provide an example?

How important is it for...

<u>Organizational/Structural factors:</u>

2b) participation in an IPE course to be implemented for all students in the core curriculum or that it takes place as a sustainable elective course?
2c) the IPE course to be financially supported?

<u>Content-based (didactic, pedagogical) factors:</u>

2d) interprofessional competencies/goals to be defined and achieved?
2e) the course to be offered to students over a longer period?
2f) good results to achieved and published?

Question 3: Despite the growing call for more interprofessional education, the current training of health professionals is largely siloed. While some universities have made changes, many have not yet done so. What do you think is hindering widespread implementation of IPE in Germany?

Follow-up questions:

3b) How likely is it that some medical schools are delaying the implementation of interprofessional education until the new medical licensing regulations, including NKLM recommendations, come into effect?
3c) A 2016 study by the Robert Bosch Foundation, which evaluated several projects, identified the curricular inflexibility of educational institutions, especially medical faculties, as a major barrier to the sustainable implementation of interprofessional education. What do you think about that?
3d) In your opinion, are there any other significant barriers?
3e) How can an interprofessional course be integrated into medical education without compromising licensure requirements? Does a model medical program offer more flexibility, so to speak, than a regular medical program?

Question 4: At the medical faculty of (name of institution), several interprofessional courses between medical students and students of other health professionals are embedded in the curriculum (name of course(s). In your opinion, which factors and conditions enabled these interprofessional courses?

Follow-up questions:

- Can you think of anything else regarding this?
- Can you provide an example?

4b) Was the decision to incorporate sustainable IPE also made to adapt the curriculum to modern evidence-based healthcare and best practice recommendations?
4c) Can it be said that people with real decision-making authority within the faculty significantly influenced the decision to invest in interprofessional education?
4d) Since when has IPE been part of your curriculum?
4e) Are the health professions participating in the IPE course(s) all located at the university?

Attachment 3 to González Blum C, Richter R, Walkenhorst U. *Interprofessional education at medical faculties in German-speaking countries – institutional challenges and enablers of successful curricular implementation: A mixed-methods study.* GMS J Med Educ. 2025;42(4):Doc45. DOI: 10.3205/zma001769

4f) Do you have an interprofessional training ward/station?
4g) Do you have an official coordinator for the organization, planning and implementation of IPE?
4h) Ultimately, implementing interprofessional education, even if it is not yet mandatory, is a conscious decision with potential benefits, but also risks. How does the medical school view the perceived benefits of implementing interprofessional teaching versus the perceived risks of implementation?

Question 5: Can you explain the biggest challenges and problems your institution has overcome in establishing IPE?

Follow-up questions:

- Can you think of anything else regarding this?
- Can you provide an example?

5b) What motivated your institution to muster the energy to take this step anyway?
5c) How great was/is the organizational effort?
5d) Were there any challenges in implementing interprofessional courses, and if so, how were they overcome?

Question 6: The widespread implementation of interprofessional education (with NKLM recommendations) will only be possible through a change of licensing regulations. However, it is unclear when exactly this change will take place. What specific recommendations can be made for interprofessional projects that are now aiming for long-term implementation in the curriculum to ensure its integration?

Follow-up questions:

- Can you think of anything else regarding this?
- Can you provide an example?

6b) If the biggest challenge is a lack of sustainable funding e.g. for a person to coordinate IPE projects?
6c) If the biggest challenge is a lack of support for interprofessional education in general?

Question 7: Would you like to add anything else regarding the topic?

Attachment 3 to González Blum C, Richter R, Walkenhorst U. *Interprofessional education at medical faculties in German-speaking countries – institutional challenges and enablers of successful curricular implementation: A mixed-methods study.* GMS J Med Educ. 2025;42(4):Doc45. DOI: 10.3205/zma001769
