# Supplementary material for: Interprofessional education at medical faculties in German-speaking countries – institutional challenges and enablers of successful curricular implementation: A mixed-methods study
Source: GMS J Med Educ. 2025 Sep 15;42(4):Doc45. doi: 10.3205/zma001769 (PMC12527387; doi:10.3205/zma001769)
Supplement: Qualitative content analysis according to Kuckartz [file JME-42-45-s-004.pdf]

Attachment 4: Qualitative content analysis according to Kuckartz

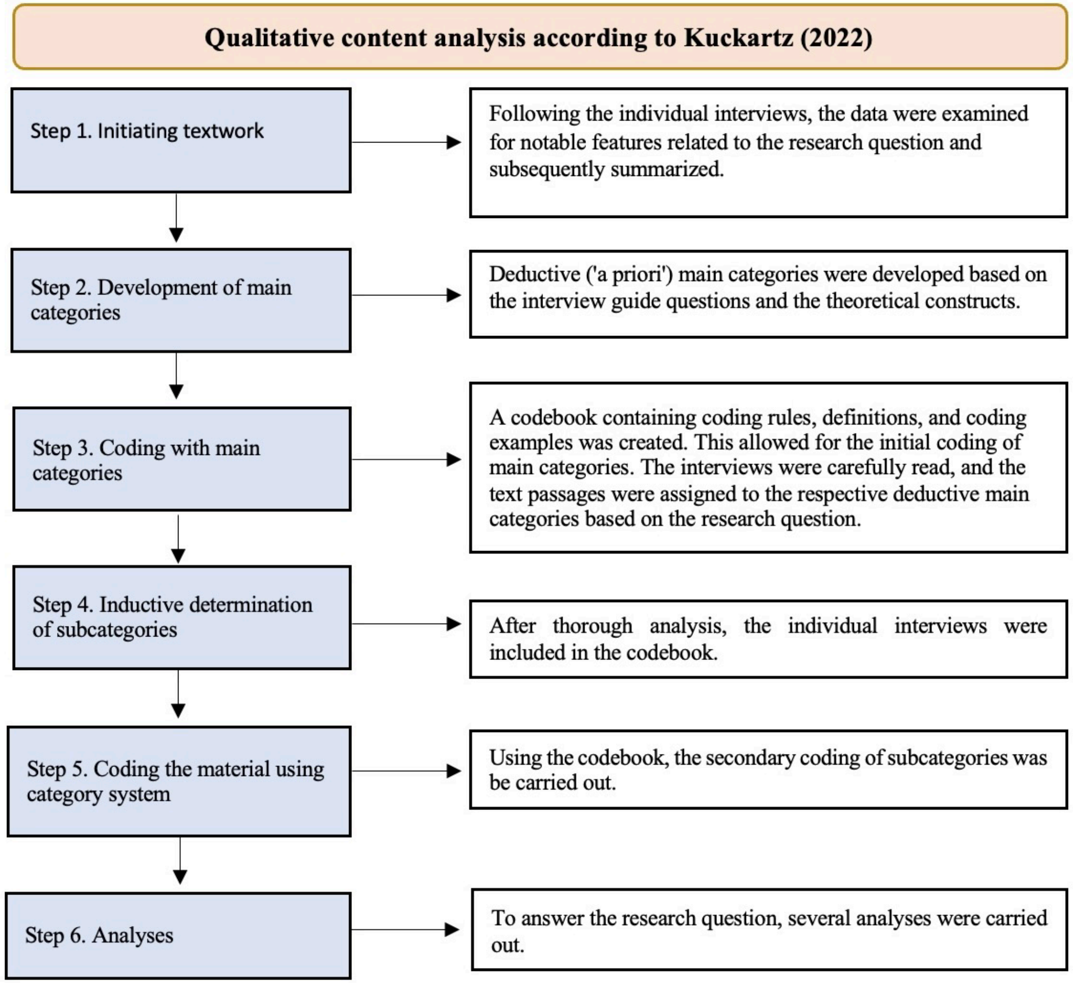

Process of the qualitative content analysis
