# Supplementary material for: Interprofessional education at medical faculties in German-speaking countries – institutional challenges and enablers of successful curricular implementation: A mixed-methods study
Source: GMS J Med Educ. 2025 Sep 15;42(4):Doc45. doi: 10.3205/zma001769 (PMC12527387; doi:10.3205/zma001769)
Supplement: Multidimensional category analysis [file JME-42-45-s-005.pdf]

Attachment 5: Multidimensional category analysis

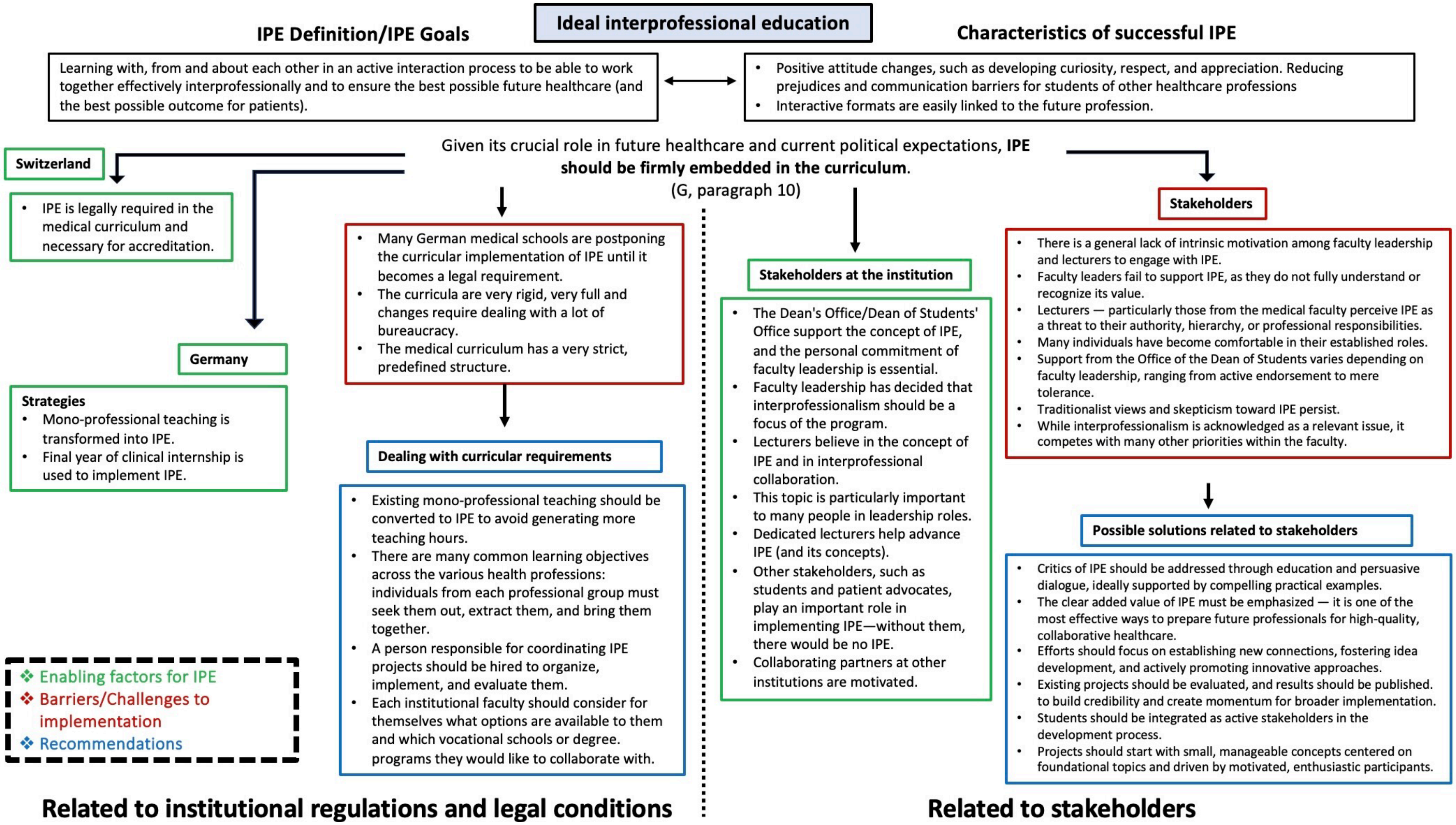

The analysis included expert opinions from seven main categories. The combined main categories **IPE Definition, IPE Goals,** and **Characteristics of Successful IPE** describe what constitutes "ideal" IPE. Regarding the differentiation of institutional regulations/legal conditions and stakeholders, the other main categories **Enabling Factors, Overcome** and **Ongoing Challenges for IPE**, as well as **Expert recommendations** for addressing these, are presented.

Attachment 5 to González Blum C, Richter R, Walkenhorst U. *Interprofessional education at medical faculties in German-speaking countries – institutional challenges and enablers of successful curricular implementation: A mixed-methods study.* GMS J Med Educ. 2025;42(4):Doc45. DOI: 10.3205/zma001769
