# Supplementary material for: Interprofessional education at medical faculties in German-speaking countries – institutional challenges and enablers of successful curricular implementation: A mixed-methods study
Source: GMS J Med Educ. 2025 Sep 15;42(4):Doc45. doi: 10.3205/zma001769 (PMC12527387; doi:10.3205/zma001769)
Supplement: Stakeholder analyses [file JME-42-45-s-006.pdf]

Attachment 6: Stakeholder analyses

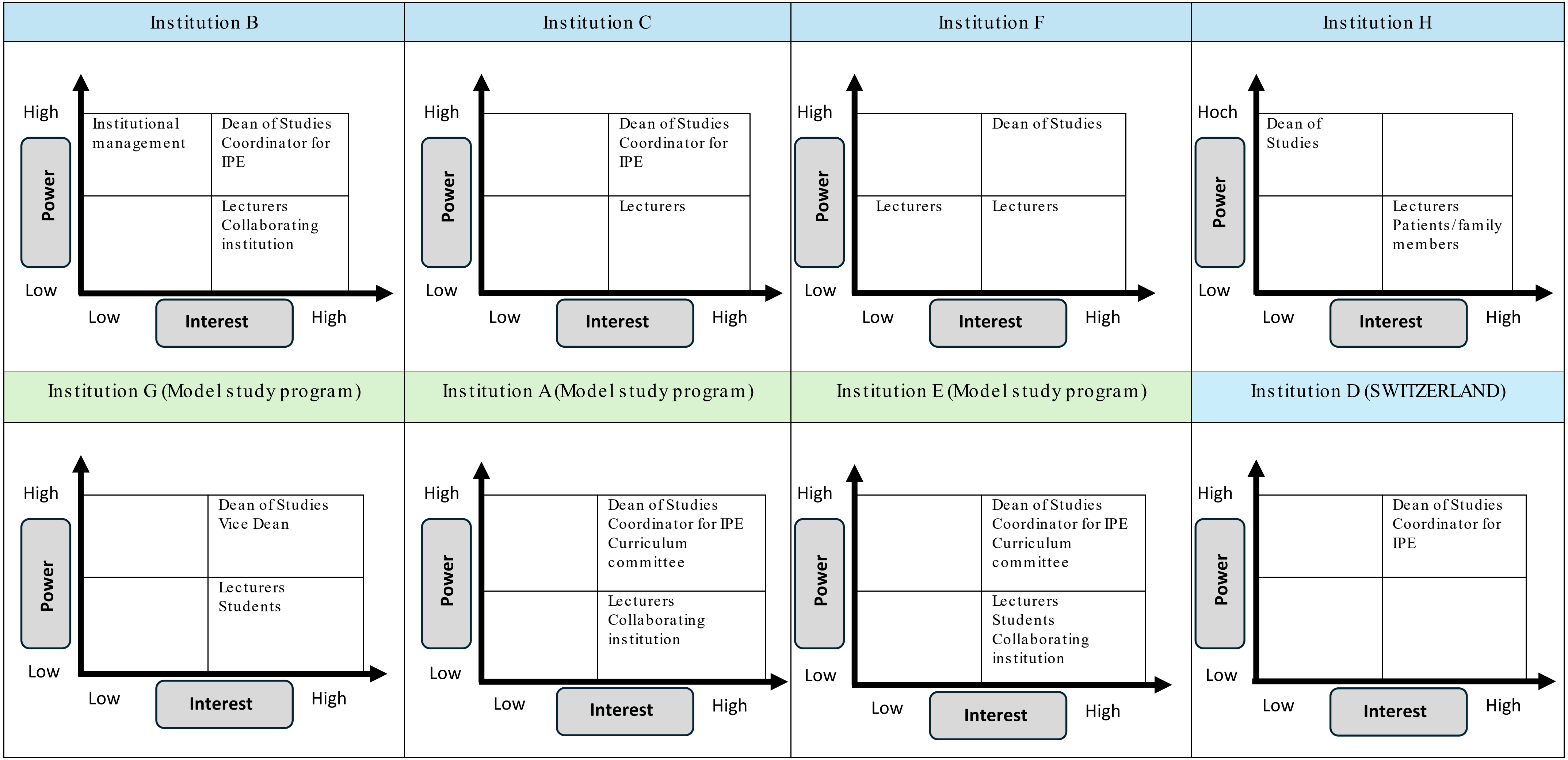

Stakeholder analyses carried out at eight medical faculties (Institutions) illustrate the different constellations of stakeholders. A stakeholder analysis was conducted for each medical faculty.

Attachment 6 to González Blum C, Richter R, Walkenhorst U. *Interprofessional education at medical faculties in German-speaking countries – institutional challenges and enablers of successful curricular implementation: A mixed-methods study.* GMS J Med Educ. 2025;42(4):Doc45. DOI: 10.3205/zma001769
